# Study on Multicellular Systems Using a Phase Field Model

**DOI:** 10.1371/journal.pone.0033501

**Published:** 2012-04-23

**Authors:** Makiko Nonomura

**Affiliations:** 1 Department of Mathematical Information Engineering, College of Industrial Technology, Nihon University, Narashino-shi, Chiba, Japan; 2 Japan Science and Technology Agency, PRESTO, Kawaguchi-shi, Saitama, Japan; University of Georgia, United States of America

## Abstract

A model of multicellular systems with several types of cells is developed from the phase field model. The model is presented as a set of partial differential equations of the field variables, each of which expresses the shape of one cell. The dynamics of each cell is based on the criteria for minimizing the surface area and retaining a certain volume. The effects of cell adhesion and excluded volume are also taken into account. The proposed model can be used to find the position of the membrane and/or the cortex of each cell without the need to adopt extra variables. This model is suitable for numerical simulations of a system having a large number of cells. The two-dimensional results of cell division, cell adhesion, rearrangement of a cell cluster, chemotaxis, and cell sorting as well as the three-dimensional results of cell clusters on the substrate are presented.

## Introduction

In order to investigate the structural patterns of cellular systems, several cell models have been reported, including the vertex dynamics model [1], [2], the center dynamics model [3], [4], and the cellular Potts model [5], [6]. Both the vertex dynamics model and the center dynamics model express cell patterns using polygons. In the vertex dynamics model, a cell or a cluster of cells is represented by a polygon formed by linking several vertices. Each vertex is driven by forces acting on it. This model has been adopted for morphogenesis in Xenopus notochords as well as cell deformation and rearrangement by applying mechanical forces [1], [7]. In the center dynamics model, a node represents a cluster of cells and receives forces from its neighboring nodes. Cell aggregation, locomotion, rearrangement, and morphogenesis in vertebrate limb buds have been investigated using this model [3], [4], [8]–[10]. Although the mechanical processes during tissue developments can be well investigated, artificial treatments are required for numerical simulations in these models based on polygons. For example, in the vertex dynamics model, cell rearrangement is realized by manually exchanging two vertices that approach each other [1]. In the center dynamics model, in order to express the cell division, it is necessary to add a new node in the vicinity of the existing node [4], [10].

In contrast, the cellular Potts model represents each cell as a cluster of grid points under the constraint of constant volume. Thus, the artificial treatments mentioned above are not required for simulations in this model. We can investigate the deformation of an individual cell in a multicellular system using this model, considering the effects of excluded volumes and adhesions of the cells. This model successfully described several biological behaviors [11]. For example, numerical calculations with regard to cell sorting, biofilm formation, and chemotactic movement have been performed [5], [6], [12], [13]. However, running the simulations requires fluctuations, and the forces between cells are not expressed directly in this model.

Therefore, we consider a new type of a model for multicellular systems, which is based on the phase field model. The effects of cell adhesion and excluded volume are taken into account. In the proposed model, the free energy is described in terms of a vector variable, the number of components of which is equivalent to the total number of cells in the system. The shape of one cell is expressed by one component of the vector variable. The time evolutions are described by a set of partial differential equations that are obtained by taking the functional derivative of the free energy. Thus, fluctuations are not required for numerical simulations. In addition, by adopting auxiliary variables that are used for calculation of the interactions between the cells, a program that consumes little computational memory can be designed. That is to say, the proposed model can be used to describe a system containing a large number of cells. The proposed model differs from previous models of multicellular systems in that the position of the cell membrane and/or cortex can also be expressed without the need to adopt extra variables because the phase boundary interface is treated as a diffuse interface of finite width using the phase field method.

The phase field model has been applied to a wide range of problems, such as crystal growth [14]–[18]. Very recently, the cell shape of the fish keratocyte has been modeled using this method, where the membrane bending force and the surface tension of the cell were considered [19]. However, to our knowledge, this is the first report applying the phase field method to the multicellular system.

## Results and Discussion

### Model Equation

We consider a multicellular system containing several types of cells and allow changes in the size and adhesive strength of each cell type. As a first step, we express the shape of one cell using the phase field method.

**Figure 1:**
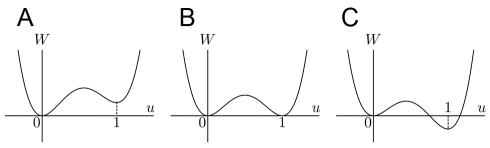
Shape of the double-well potential *W*(*u*). The parameters are set as 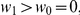 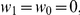 and 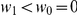 in Panels A, B, C, respectively.

The phase field model is based on the following Ginzburg-Landau free energy:
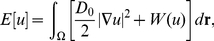
(1)where 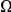 denotes the area of the system, and the coefficient 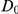 is a positive constant. The variable 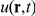 is an order parameter referred to as the phase field, where 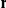 is the position, and *t* is the time. The function 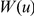 is given as
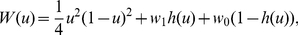
(2)where the function 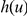 is defined as



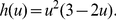
(3)Equation 2 describes a double-well potential which has local minimums at 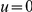 and 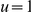 under the condition 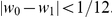 As shown in Figure 1, the depths of the wells are controlled by the constants 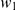 and 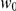 which correspond the free energy densities for the phases described by 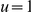 and 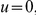 respectively.

By taking the functional derivative of Equation 1 with respect to 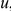 the time evolution of 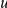 is derived as follows:
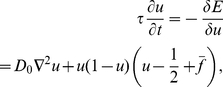
(4)where 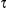 is a positive constant and 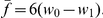 Equation 4 guarantees the monotonic decrease in the free energy. Equation 4 is referred to as the Allen–Cahn equation in the field of materials science and is known for having a smooth front solution connecting the regions 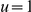 and 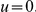 The Allen–Cahn equation can easily be solved in one dimension as 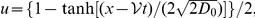 where the front velocity 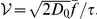 This means that the front moves such that the region of 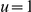 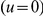 expands if 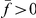 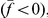 i.e., 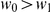 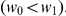


In order to describe a cell shape by the variable 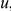 the constraint of the constant volume of 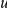 should be included in Equation 4. When the volume of the region in which 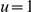 is denoted by the function 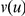, it is easy to consider this constraint by replacing the constant 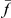 in Equation 4 with the following function 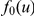:
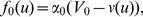
(5)as
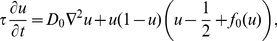
(6)where the coefficients 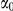 and 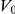 are positive constants. As discussed in the above paragraph, it is obvious that Equation 6 expresses the region of 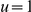 expands (shrinks) until 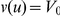 when 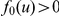 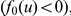 i.e., 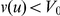 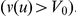


By choosing the form of the function 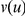 as
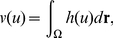
(7)we also obtain the free energy form for Equation 6 as follows:



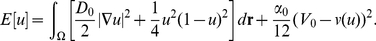
(8)Note that Equation 7 can be regarded as the volume of the region in which 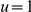 because 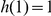 and 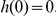 Therefore, the last term of Equation 8, which is newly added, expresses the constraint of the constant volume of *u* since it has a minimum at 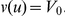


**Figure 2:**
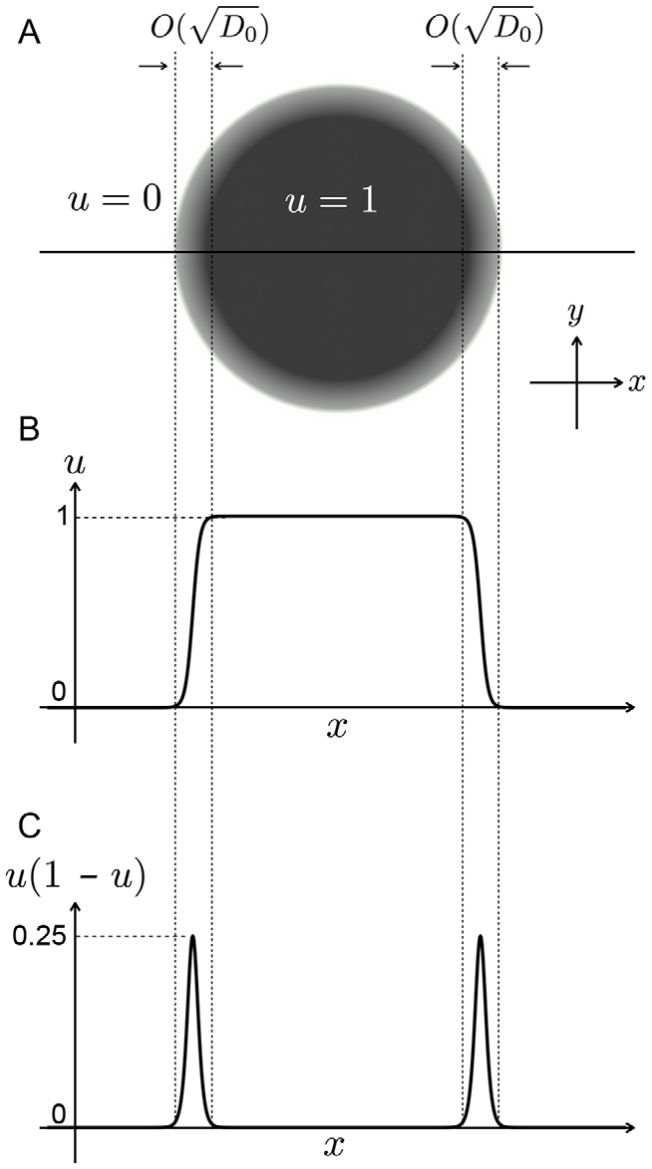
Shape of the phase field *u*. The integral of *u* over **r** is set to be maintained. Panel A: top view. Panels B and C: profiles of *u* and 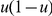 at the centerline in Panel A, respectively.

As shown in Figure 2, the region of 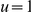 takes the form of a circle in two dimensions and a sphere in three dimensions in the steady state. Thus, the shape of the cell in the simplest case can be described by a single-order parameter 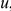 such that 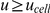 in the region with the cell (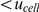 in the region not taken up by the cell) with a constant 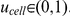 Based on the fact that 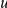 has an interface with a thickness on the order of 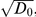 the cell cortex can also be expressed as a function of *u*, e.g., 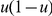 (see Figure 2C).

In order to describe the multicellular system, a vector variable 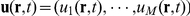 is considered, where 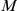 is the total number of cells in the system. The component 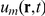 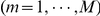 describes the shape of the *m*-th cell. We also use the variable 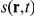 to represent the shape of substances interacting with the cells, such as the wall, the substrate, and the extracellular matrix.

The model free energy for the multicellular system is written as
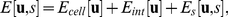
(9)where 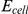 determines the shape of the cell, 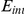 describes the interactions between each cell, and 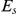 expresses the interactions between the cells and substances external to them. The form of 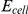 is obtained by modifying Equation 8 using the vector variable as follows:
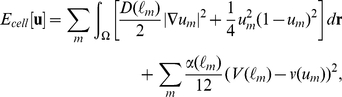
(10)where 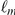 is the cell type of the m-th cell. The coefficients 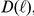 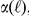 and 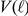 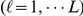 are positive constants, where L is the total number of cell types in the system. As discussed previously, Equation 10 indicates that the thickness of the cell interface is on the order of 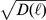 and that the speed at which the volumes of the type-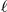 cells approach the target volume 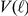 is controlled by the value of 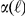. That means 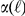 determines the cell size growth. Here, 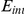 can be presented in the following form:
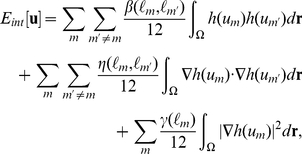
(11)where 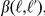 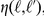 and 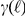 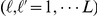 are positive constants. The first term on the right-hand side of Equation 11 represents the effect of the excluded volume by increasing the energy if the cells overlap, whereas the second term represents the effect of cell adhesion by decreasing the energy if the cell cortices overlap. This adhesion term becomes negative in the region in which cell adhesion occurs. In order to prevent divergence due to this adhesion term, we introduce the third term on the right-hand side of Equation 11 with the condition whereby 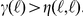 Similarly, the interaction between cells and substances external to the cells is expressed as follows:
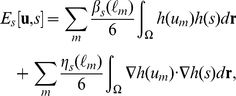
(12)where 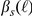 and 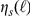 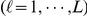 are positive constants.

**Figure 3:**
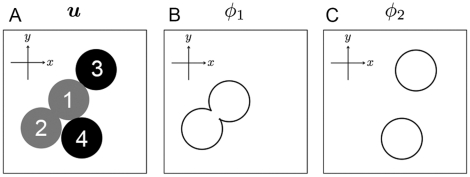
Schematic diagram of 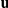 and 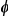. In Panel A, type-1 (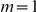 and 2) and type-2 (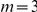 and 4) cells are represented by gray and black circles, respectively. The contours of 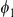 and 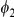 are indicated by curved lines in Panels B and C, respectively.

Taking the functional derivative of Equation 9 with respect to 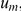 the following time evolution equations are obtained:
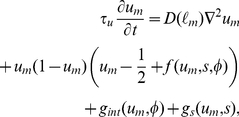
(13)

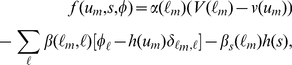
(14)

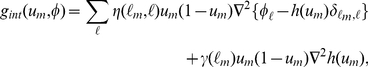
(15)

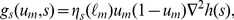
(16)where 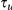 is a positive constant, and 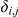 is the Kronecker delta, which is 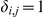 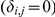 if 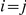 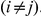 The vector variable 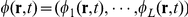 is an auxiliary variable that is defined as follows:



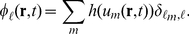
(17)As shown in Figure 3, the region occupied by the type-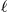 cells can be identified by 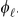


Note that the interaction terms in Equation 13 are not written explicitly in terms of the variables 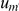 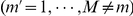 but are instead written in terms of the auxiliary variable 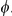 As discussed in Methods, the introducing 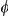 is very useful for the simulation of a system having a large number of cells. The components 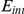 and 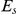 can also be presented in terms of 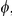 as follows:
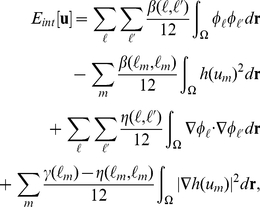
(18)

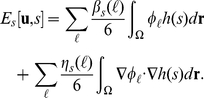
(19)


We adopted the second term on the right-hand side of Equation 14 and the first term on the right-hand side of Equation 15 to express the excluded volumes and the cell adhesions, respectively, because these terms are the simplest among the several alternatives, which can be written in terms of 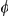 both in the time evolution equation for 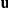 and the component 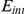.

### Numerical Simulation

**Figure 4:**
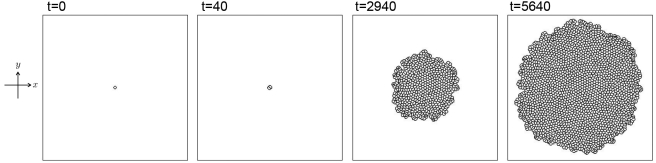
Two-dimensional result of cell division. All cells are set to be of the same type 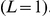 Contour plots of 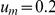 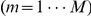 are indicated by the black curves. The number of cells 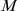 is increased by cell divisions: 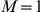 at 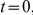 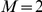 at 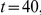 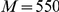 at 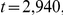 and 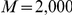 at 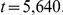 The other parameters are set as follows: size of the simulation box 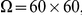 size of the spatial grid 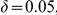 time increment 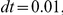 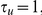 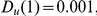 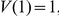 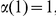 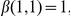 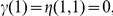 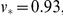 and 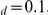

Figure 4 shows the result of cell divisions in two dimensions. It is well known that the spindle positioning plays an important role in the stage of deciding the plane of cell division [20]–[22]. However, we consider here the simplest rule for cell division as the first step. The rule imposed here is that when the volume 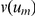 of the *m*-th cell become larger than 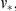 where 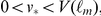 the *m*-the cell divides into two cells, 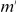 and 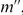 as
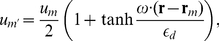
(20)

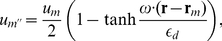
(21)where 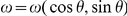 and 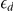 is a positive constant. The angle 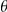 is taken randomly. Using this rule, the number of cells increase in time. Starting with the data in which a circular cell is located at the center of 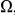 the number of cells become 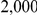 at 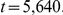 For simplicity, the number of cell type is one, 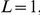 and the cell adhesion is not considered, 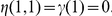 It is noted that the scale of time 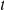 can be determined by comparison with the experimental data of the cell cycle.

**Figure 5:**
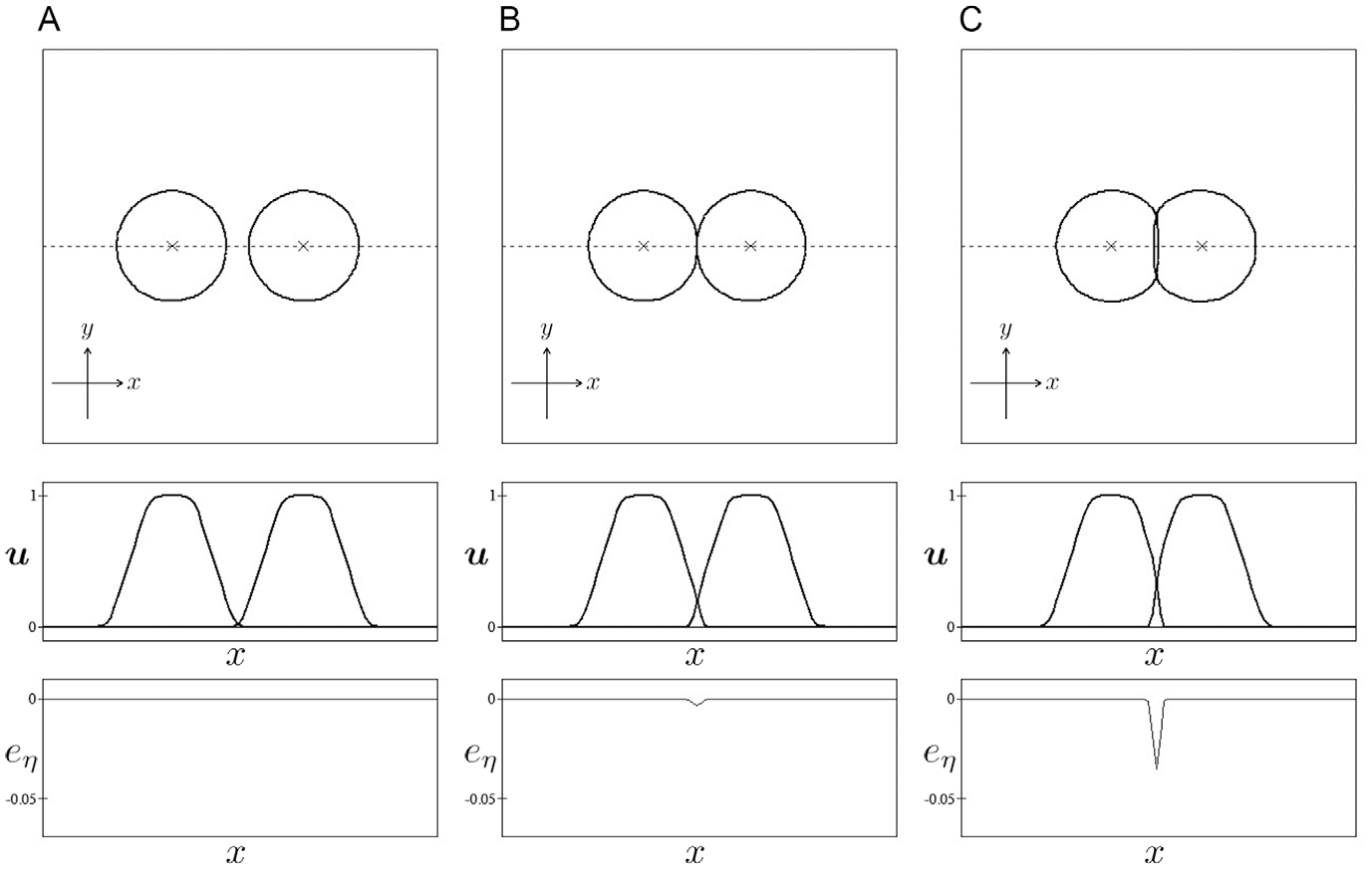
Two-dimensional results of cell adhesions. The case of two cells 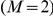 of the same type 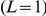 is considered. Numerical calculations were performed with 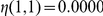 in Panel A, 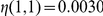 in Panel B, and 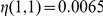 in Panel C. The top row shows contour plots of 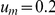 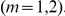 The × symbol indicates the centers of cells. The middle and bottom rows show the profiles of 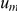 and 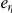 along the dotted line shown in the top row. The size of the simulation box is 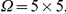 and the size of the spatial grid is 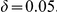 The time increment is 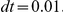 The remaining parameters are set as follows: 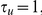 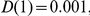 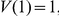 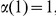 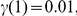 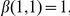 and 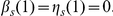

Figure 5 shows the numerical results for two cells of the same type, i.e., 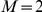 and 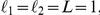 with different adhesion strengths. The curves in the top row of the graphs indicate the contour lines of 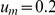 (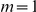 and 2), and the 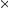 symbols indicate the positions of the centers of the cells 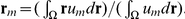 The variable 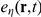 is given as follows:
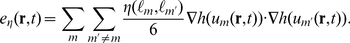
(22)


The integral over **r** of 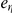 is identical to the second term on the right-hand side of Equation 11. The 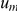 and 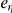 profiles along the dotted line in the top row have been plotted in the middle and bottom rows of the graphs, respectively. Since 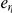 has a non-zero value only in regions in which cell adherence occurs, 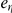 is an indicator of locations at which cell adherence occurs. Initially, the distance between the centers of cells is set to 1.400. After a sufficiently long simulation time 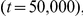 the two cells move closer to each other as the value of 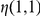 increases, such that the distances between the cell centers are 1.574 in the case of Panel A with 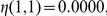 1.280 in the case of Panel B with 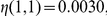 and 1.107 in the case of Panel C with 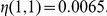


**Figure 6:**
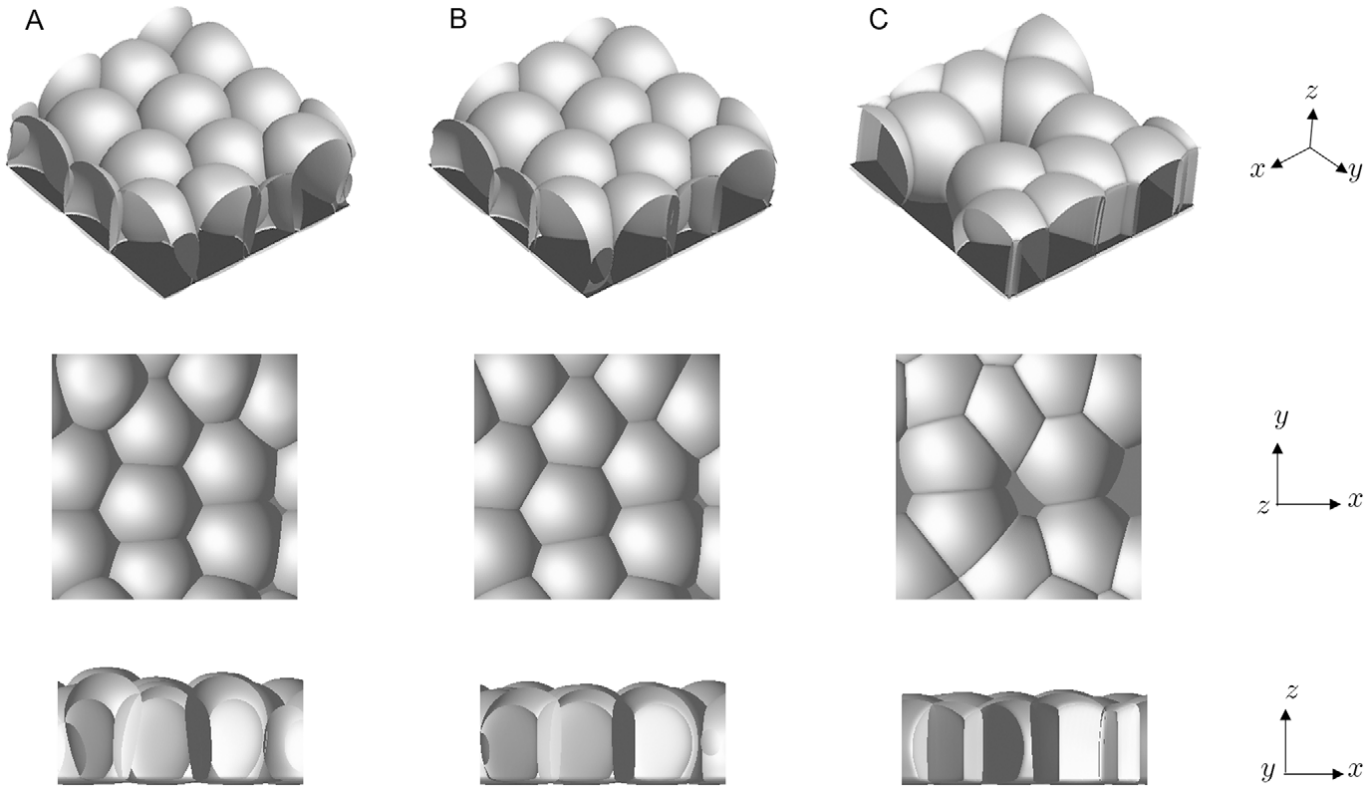
Three-dimensional results of cell adhesions on the substrate. The case of 10 cells 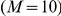 of the same type 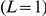 is considered. Numerical calculations were performed with 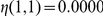 in Panel A, 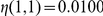 in Panel B, and 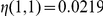 in Panel C. Light and dark gray surfaces are contour plots of 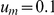 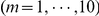 and 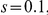 respectively. The diagonal, top, and side views for each result are shown in the top, middle, and bottom rows, respectively. The size of the simulation box is 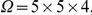 and the size of the spatial grid is 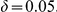 The time increment is 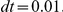 The remaining parameters are set as follows: 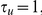 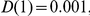 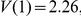 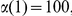 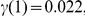 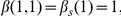 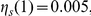 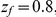 and 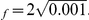

Figure 6 shows snapshots of three-dimensional simulations at 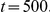 The solid substrate is introduced by setting the variable *s* as 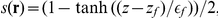 where 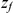 and 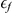 are positive constants. The light gray surfaces are contour plots of 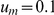 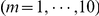 and the dark gray surfaces represent contour plots of 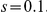 We set 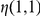 as 0.0000, 0.0100, and 0.0219 for the simulations shown in Panels A, B, and C, respectively, where the other parameters are the same for all cases. If the cell adhesions are weak, the cells push against each other, and their positions are determined as shown in Panel A. On the other hand, for the case in which the cell adhesions are sufficiently strong, the cell positions are decided by the pulling force between cells, and the surface of the cell layer becomes flat, as shown in Panel C.

**Figure 7:**
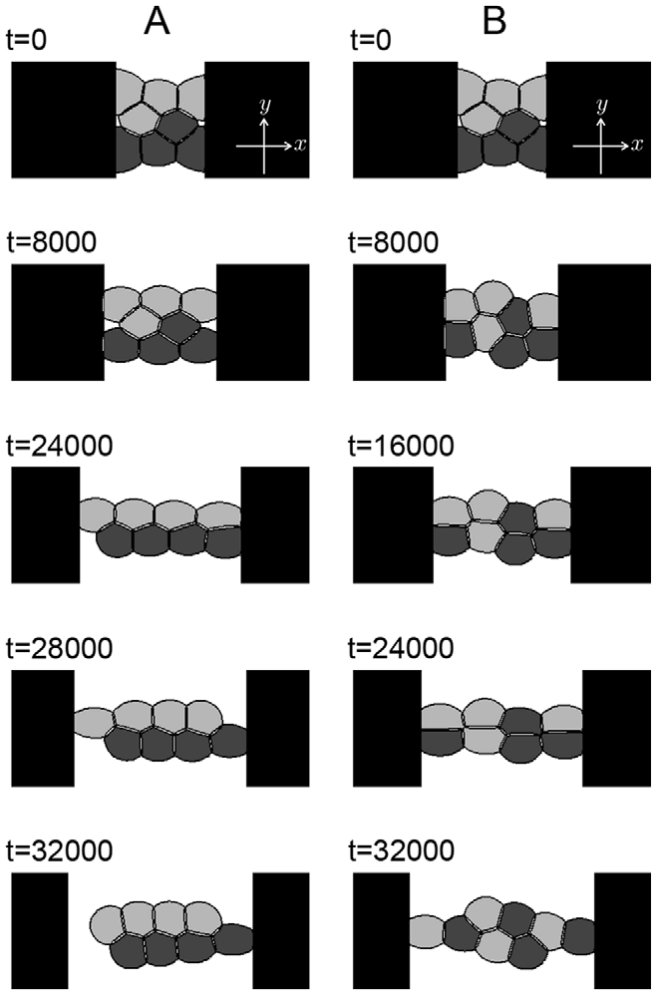
Two-dimensional results of cell deformation and rearrangement in a cluster. The cluster is composed of eight cells 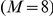 of two types 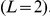 Light and dark gray areas represent the region of 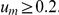 Light gray areas indicate the locations of type-1 cells, and dark gray areas indicate the locations of type-2 cells. Black areas represent the walls 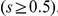 Numerical calculations were performed with 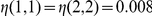 and 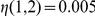 in Panel A and 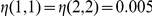 and 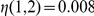 in Panel B. The left and right walls are assumed to move at a uniform velocity, 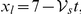 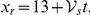 and 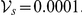 The size of the simulation box is 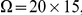 and the size of the spatial grid is 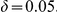 The time increment is 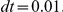 The remaining parameters are set as follows: 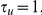 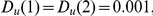 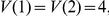 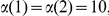 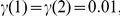 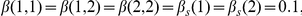 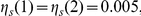 and 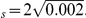

**Figure 8:**
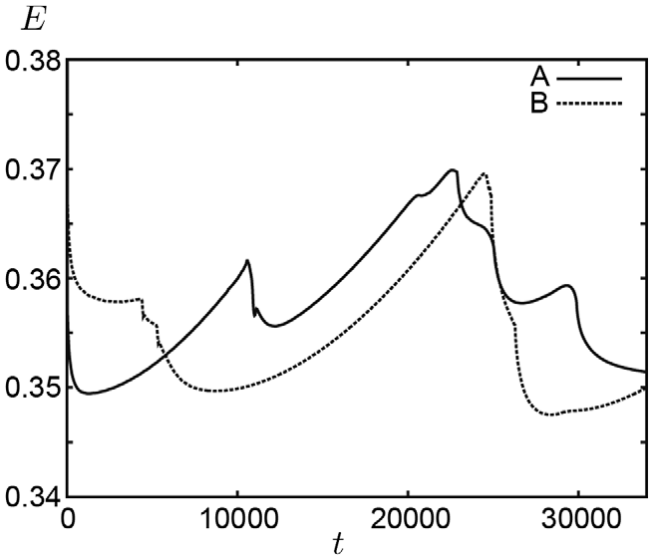
Plots of the total energy *E* with respect to time. The solid line shows the results for Figure 7A, and the dotted line shows the results for Figure 7B.

Figure 7 shows the numerical results for cell deformation and rearrangement. A cell cluster of 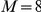 and 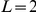 is sandwiched between two walls that move at a constant speed. In this calculation, considering the variable *s* as an order parameter that corresponds to the walls, the time evolution of *s* is calculated as 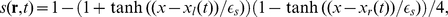 where 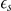 is a positive constant. The locations of the left and right walls are denoted as 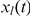 and 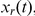 respectively. Panel A shows the results for the case in which the adhesion strength between cells of the same type is stronger than that between cells of different types (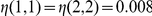 and 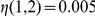), whereas Panel B shows the results for the opposite case 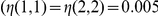 and 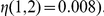 Light gray, dark gray, and black areas represent the positions of the type-1 cells, the type-2 cells, and the walls, respectively. Cells adhering to the walls are stretched by the moving walls, causing the cells to be deformed and rearranged. Cells that are rearranged as weakly adhered cells detach first. In Panel A, the cells detach from the left wall at approximately 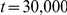 and relax to almost their original shape at 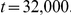 The time evolution of the total energy *E* is plotted in Figure 8. The solid line shows the results for Panel A of Figure 7, and the dotted line shows the results for Panel B of Figure 7. There is no monotonic decrease in total energy because the walls stretch the cell clusters. Comparison of Figures 7 and 8 reveals that the energy decreases significantly when cell rearrangement occurs.

**Figure 9:**
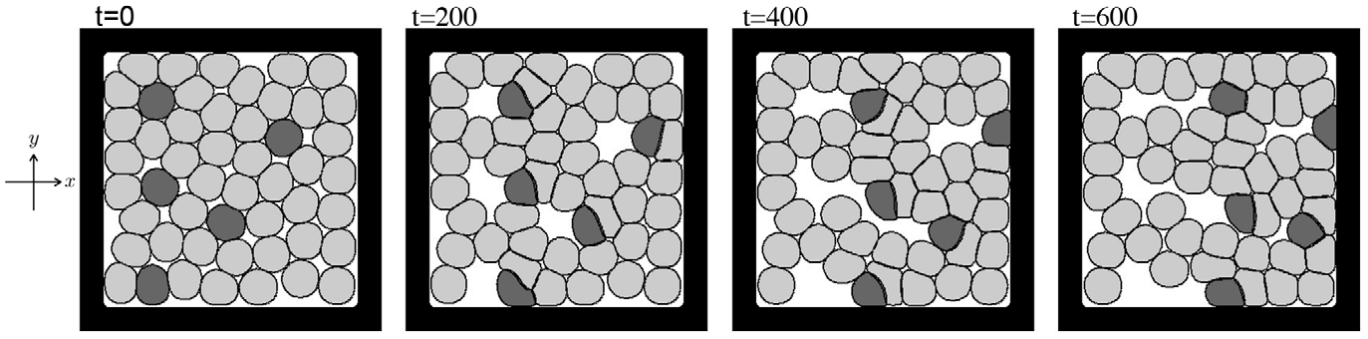
Two-dimensional result of chemotactic movement of cells. The case of fifty cells 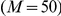 of two types 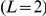 is considered. Light gray (dark gray) areas indicate the region of 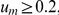 for the case in which the *m*-th cell is a type-1 (type-2) cell. Black areas represent the walls 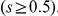 Numerical calculation was performed with 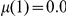 and 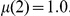 The other parameters are set as follows: size of the simulation box 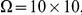 size of the spatial grid 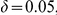 time increment 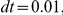 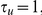 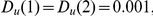 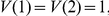 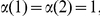 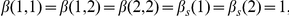 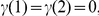 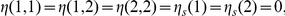 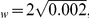 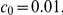 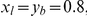 and 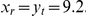

Finally, we show that the cell movements such as the chemotactic movement and the random movement can also be incorporated into the proposed model. The chemotactic movement of the cell can be described by adding a new term, such as 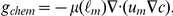 to the right-hand side of Equation 13, where the variable 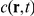 is the chemical concentration in extracellular regions. The parameter 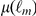 indicates the sensitivity of the *m*-th cell to the gradient of *c*. Figure 9 shows the time evolution of a system with cells having chemotaxis. Light gray and dark gray represent type-1 and type-2 cells, respectively. In this case, we consider the variable *s* as an order parameter that corresponds to the wall. The fifty cells are surrounded by the unmoving wall defined as 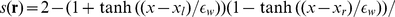 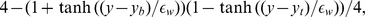 where 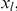 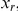 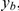 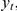 and 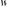 are positive constants. By setting 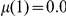 and 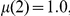 it is assumed that the only type-2 cells can sense the gradient of the chemical concentration *c*. For simplicity, cell adhesion is not considered and the form of *c* is assumed not to be affected by 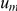 or *t* and is taken as 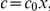 where 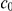 is a constant. It is found numerically that type-2 cells move toward the *c*-rich region by pressing against type-1 cells.

**Figure 10:**
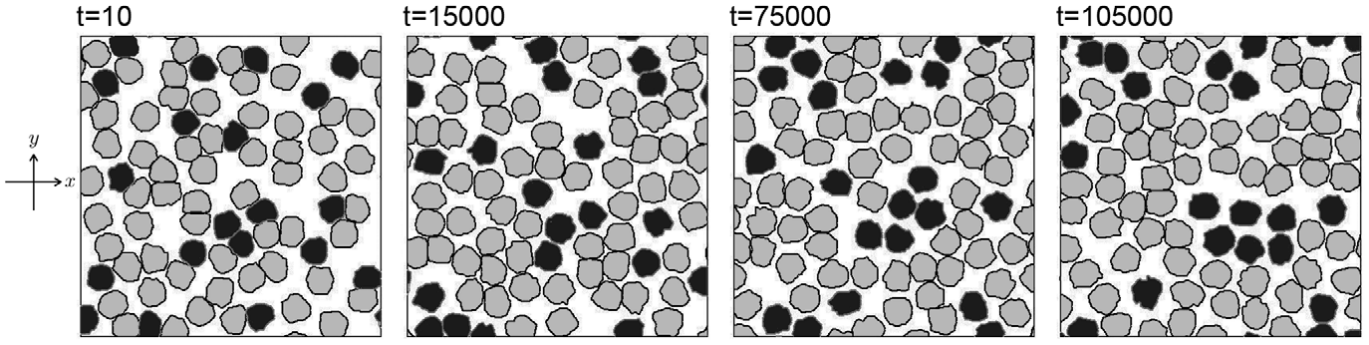
Two-dimensional result of cell sorting. The case of eighty cells 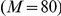 of two types 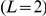 is considered. The number of the type-2 cells is 20. Light gray (dark gray) areas indicate the region of 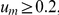 for the case in which the *m*-th cell is a type-1 (type-2) cell. The other parameters are set as follows: size of the simulation box 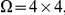 size of the spatial grid 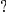 time increment 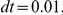 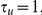 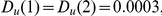 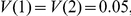 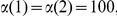 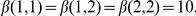 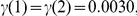 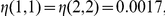 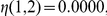 and 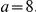

The random movement of cells can also be incorporated into the proposed model by adding a new term, such as 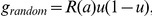 to the right-hand side of Equation 13. The function 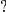 indicates an uniform random number from 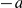 to 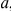 where 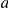 is a positive constant. Figure 10 shows the result of cell sorting which has been known to require both of the random movement of cells and the cell adhesion [5], [6], [12]. We obtained that differential adhesion with fluctuation leads the sorting of a mixture of two types of cells as reported in Ref. [6].

### Conclusion

We proposed a new type of cell model based on a phase field model, including the effects of excluded volumes and cell adhesions. To our knowledge, this is the first study to apply the phase field model to multicellular systems. We succeeded to make a skillful method for reduction of the computational memory and simulation time using the auxiliary variable 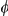 as discussed in Methods.

The proposed model is based on a concept similar to the cellular Potts model, but the time evolutions of cell shapes in the proposed model differ from those in the cellular Potts model. In the cellular Potts model, the time evolutions of the spins are computed by the Monte Carlo method, and thus the fluctuations are required for the time evolution. On the other hand, the time evolution equations in the present model are written in the form of partial differential equations, whereby fluctuations are not necessary in order to run the simulations. In addition, the proposed model is thought to be more appropriate for investigating problems in which a small volume variant must be accounted for, because the proposed model is continuous in any parameter.

Since the cell shapes are represented by interfaces of finite thickness, the proposed model has the potential to be applied to the investigation of not only shape changes due to interactions between cells (Figures 5 and 6) and rearrangements of cells in clusters (Figure 7) but also phenomena requiring knowledge of the position of the cell membrane and/or cortex. It is easy to incorporate additional cell behaviors such as chemotaxis (Figure 9) and random movement (Figure 10) into the proposed model by adding corresponding terms.

The proposed model can express the time evolution of changes in cell shape due to the interactions between cells, cell differentiation by changing the cell type, cell size growth, cell movement, and cell death by deleting the corresponding component of 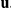 Thus, this model may well provide a useful tool for approaching the problem of morphogenesis, although this remains a subject for future study in order to estimate the parameters as well as the time scale by comparison with earlier models and with experimental data. Considering both of the adhesion and the random movement of cells, we will investigate the chemotactic movement by using the proposed model and compare the obtained results to those reported in Ref. [12] as the next step. We also plan to include the cell division, in the process of which the cortex of the dividing cell is known to be important as well as the spindle positioning [20]–[22], in the present model and to approach the problem of morphogenesis.

## Methods

### Numerical Implementation

In order to rapidly simulate a system having numerous cells, it is important to design a program that does not consume a large amount of computational memory and to increase the simulation speed. These two requirements are easily satisfied because Equation 13 is not written explicitly in terms of 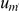 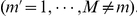 Once 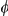 is obtained for each time step, the time evolution of 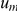 can be computed independent of 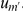 Such a program is fully compatible with parallel computation. Moreover, the shape of the *m*-th cell can be obtained by computing the equation for 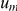 within the small region 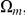 which covers the region of 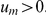 This reduces the computational memory and increases the simulation speed. The position 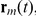 which indicates the center position of 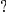 measured for the entire system, must be moved along with the movement of the center position of the *m*-th cell. Since 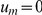 is realized outside the region 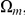 the Dirichlet boundary condition must always be set for the small region 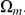 In this paper, the periodic boundary conditions are imposed on the boundary of 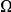 throughout the simulations.

The estimation of the required memory is described below. Since the number of cell types 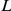 is generally much smaller than the number of cells 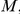 the memory increase by introducing 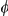 becomes smaller than the memory decrease by computing 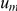 within the small region 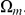 For simplicity, we assume that each of the cells has the same volume, i.e., 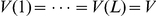 and that the entire system is covered by the cells, i.e., 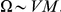 Then, the computational memories for 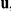 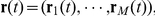 and 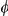 are roughly estimated as 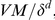 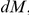 and 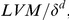 respectively, where 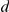 is the spatial dimension and 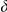 is the size of the spatial grid. Therefore, the total memory required to compute Equation 13 using 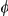 is linearly dependent on 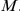 On the other hand, in order to compute cell-cell interactions without using 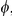 the value of 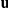 must be preserved over the entire region 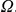 Then, the computational memories for solving Equation 13 increase by 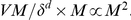 These estimations reveal that the introduction of 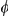 is very useful for computation in the case of a system that contains a large number of cells, even in three dimensions.

In fact, we have checked the required memory with and without adopting auxiliary variable 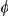 by changing the maximum number of cells, 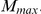 All other settings are the same for Figure 4. We used MacPro (3.2 GHz Quad-Core Intel Xeon, 32 GB 800 MHz DDR2 FB-DIMM) for this purpose. If the auxiliary variable 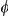 is not considered, Equation 13 should be solved in the whole region 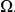 In this case, a segmentation fault occurs at start of simulation when 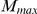 is set to be larger than 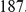 As discussed above, if the auxiliary variable 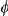 is considered, the shape of the *m*-th cell can be obtained by computing the equation for 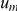 within the small region 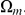 In the case of 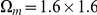 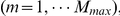 the maximum number of cells 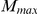 can be set to be 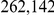 at a maximum without a segmentation fault. These results support our claim which is that the introducing the auxiliary variable 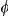 is very useful for computation in the case of a system with numerous cells.
